# Quantifying postprandial glucose responses using a hybrid modeling approach: Combining mechanistic and data-driven models in The Maastricht Study

**DOI:** 10.1371/journal.pone.0285820

**Published:** 2023-07-27

**Authors:** Balázs Erdős, Bart van Sloun, Gijs H. Goossens, Shauna D. O’Donovan, Bastiaan E. de Galan, Marleen M. J. van Greevenbroek, Coen D. A. Stehouwer, Miranda T. Schram, Ellen E. Blaak, Michiel E. Adriaens, Natal A. W. van Riel, Ilja C. W. Arts

**Affiliations:** 1 TiFN, Wageningen, Netherlands; 2 MaCSBio Maastricht Centre for Systems Biology, Maastricht University, Maastricht, Netherlands; 3 Department of Human Biology, NUTRIM School of Nutrition and Translational Research in Metabolism, Maastricht University, Maastricht, Netherlands; 4 Division of Human Nutrition and Health, Wageningen University, Wageningen, Netherlands; 5 Department of Biomedical Engineering, Eindhoven University of Technology, Eindhoven, Netherlands; 6 CARIM School for Cardiovascular Diseases, Maastricht University, Maastricht, Netherlands; 7 Department of Internal Medicine, Maastricht University Medical Center, Maastricht, Netherlands; 8 MHeNs School for Mental Health and Neuroscience, Maastricht University, Maastricht, Netherlands; 9 Heart and Vascular Center, Maastricht University Medical Center, Maastricht, Netherlands; Faculty of Medicine, University of Belgrade, SERBIA

## Abstract

Computational models of human glucose homeostasis can provide insight into the physiological processes underlying the observed inter-individual variability in glucose regulation. Modelling approaches ranging from “bottom-up” mechanistic models to “top-down” data-driven techniques have been applied to untangle the complex interactions underlying progressive disturbances in glucose homeostasis. While both approaches offer distinct benefits, a combined approach taking the best of both worlds has yet to be explored. Here, we propose a sequential combination of a mechanistic and a data-driven modeling approach to quantify individuals’ glucose and insulin responses to an oral glucose tolerance test, using cross sectional data from 2968 individuals from a large observational prospective population-based cohort, the Maastricht Study. The best predictive performance, measured by *R*^2^ and mean squared error of prediction, was achieved with personalized mechanistic models alone. The addition of a data-driven model did not improve predictive performance. The personalized mechanistic models consistently outperformed the data-driven and the combined model approaches, demonstrating the strength and suitability of bottom-up mechanistic models in describing the dynamic glucose and insulin response to oral glucose tolerance tests.

## Introduction

Maintaining glucose homeostasis within a narrow physiological range is essential for normal body functioning. When glucose levels are elevated (i.e. following meal intake), the hormone insulin is secreted from pancreatic *β*-cells to promote glucose uptake in peripheral organs and suppress hepatic glucose production [1]. In individuals with insulin resistance there is an impairment in the uptake of glucose by the insulin-dependent tissues (i.e. muscle, and adipose), and in the suppression of hepatic glucose production. The increased demand on pancreatic *β*-cell to produce and secrete more insulin may eventually lead to *β*-cell dysfunction, which is the key factor in the development of type 2 diabetes mellitus (T2DM), and is characterised by persistent hyperglycemia [2, 3]. The pathophysiology of T2DM is known to be heterogeneous; with inter-individual differences in the severity of insulin resistance and progression of *β*-cell dysfunction [4]. Physiology-based mathematical models of the glucose-insulin regulatory system have long been used to provide qualitative and quantitative information on relevant physiological processes governing postprandial glucose and insulin dynamics [5–7]. The Eindhoven-Diabetes-Education simulator (eDES) is a comparatively simple model of human insulin-regulated glucose metabolism describing only the crucial reactions involved in postprandial glucose regulation through a system of coupled differential equations [7, 8]. The reactions described by this model are regulated by rate parameters, which can be estimated from postprandial glucose and insulin time-series data. In our previous work, we successfully applied a parsimonious eDES model, estimating only 4 parameters governing gastric emptying, endogenous insulin secretion and insulin-dependent glucose disposal into tissues, to quantify the postprandial glucose and insulin responses following the intake of an oral glucose load in a large population of overweight or obese but otherwise healthy individuals [9]. Our results showed that the majority of the individuals’ responses were accurately estimated with the eDES model, nevertheless we have identified some cases where the mechanistic model struggled to capture the response. While, the intra-individual variability in the postprandial responses can be largely explained by the mechanisms of glucose regulation encoded in the eDES model, it is known that other factors such as diet, physical activity, sleep and stress may affect glucose regulation [10, 11]. However, such factors are not directly included in the eDES model and their implementation within the mechanistic model may be inconvenient; requiring excessive experimentation.

In addition to physiology-based mathematical models, the application of data-driven prediction models have gained substantial interest in diabetes research, providing insight into factors contributing to, as well as predicting glucose responses to nutrient intake [12–15]. Such approaches aim to exploit the large amounts of heterogeneous data available, to find informative patterns in a data-driven way. Notably, Zeevi and colleagues [15] have shown that a machine-learning model trained on a wide variety of phenotypic information was able to accurately predict the magnitude of postprandial glucose excursions. While mechanistic models of the glucose regulatory system describe the change in glucose (and insulin) levels according to known physiological phenomena, they are limited in their scope and accuracy by the understanding of the underlying physiology as well as the availability of invasive measurements during model building. In comparison, data-driven models allow a convenient framework to integrate diverse data that may have relevance in glucose regulation without the need for a causal understanding. Recently, the bottom up and top down modeling strategies outlined above have been successfully combined to improve simulation accuracy in the field of systems biology [16–18]. Here, we combine a mechanistic model with a data-driven model to identify factors predictive of inter-individual differences in glucose and insulin dynamics following an oral glucose tolerance test (OGTT) in a large group (n = 2968) of individuals with various glucometabolic status (including normal glucose metabolism (NGM), prediabetes, as well as T2DM) participating in a population-based cohort study (The Maastricht Study) [19].

The aim of the present study is to investigate the predictive performance (explained variance, error of prediction) in simulating the postprandial glucose and insulin levels following an OGTT in individuals using the mechanistic eDES model, a data-driven model, and a hybrid combination of the two. In addition, we will compare the predictive performance between the various models and evaluated the factors underlying the inter-individual differences in the responses as derived from the models.

## Materials and methods

### Data

Data from The Maastricht Study, an observational prospective population-based cohort study [19] was used in this work. Briefly, The Maastricht Study focuses on the etiology, pathophysiology, complications, and comorbidities of T2DM, and is characterised by an extensive phenotyping approach. Individuals aged between 40 and 75 years and living in the southern part of the Netherlands were eligible for participation. Participants were recruited through mass media campaigns and from mailings through the municipal registries and the regional Diabetes Patient Registry. Known T2DM status was used in stratifying the recruitment process for efficiency. The present report includes data from the first 3451 participants who completed the baseline survey between November 2010 and September 2013. All examinations were performed within a three-month time window; the OGTT and vascular measurements were performed during different research visits. The study has been approved by the institutional medical ethical committee (NL31329.068.10) and the Minister of Health, Welfare and Sports of the Netherlands (Permit 131088–105234-PG). All participants gave written informed consent.

#### Oral glucose tolerance test

Following an overnight fast, participants underwent a standardized two-hour 75 g oral glucose tolerance test (OGTT) in order to determine glucose metabolism status [19]. Blood samples were taken under fasting conditions (t = 0) and 15, 30, 45, 60, 90 and 120 minutes after ingestion of the glucose drink in which plasma glucose and insulin concentrations were determined. Individuals relying on external insulin did not undergo the OGTT. Furthermore, individuals with more than two missing samples or missing samples at baseline (t = 0) or 2 hour post-load were excluded from the analysis.

#### Deep phenotyping features

A selection of health-related features from The Maastricht Study were used in order to provide a holistic picture of the individuals’ health state. These features pertain to health behavior (e.g. diet, physical activity, smoking), cardiovascular health, musculoskeletal health, metabolic and demographic characteristics, body composition, and biomarkers. In total 49 features were selected by a set of co-authors who are experts in the field of metabolism/diabetes. The continuous variables were transformed to zero mean and unit variance, while the categorical variables were dummy coded prior to modeling. Details about the measurements can be found in [19]. A complete list of the features used in this work is provided in the S1 Appendix of the Supplementary Material.

### Computational modeling of glucose regulation

In the present study, a variety of modeling scenarios were explored, including mechanistic models, data-driven models, and a hybrid combination of both models in order to predict the postprandial concentrations of glucose and insulin after an OGTT.

#### Eindhoven Diabetes Education Simulator

The Eindhoven Diabetes Education Simulator (eDES, version 2.0) published by Maas and colleagues [7, 8] was employed in this study. The eDES model is a physiology-based mathematical model describing the glucose regulatory system in healthy people and people with type 1 and type 2 diabetes. The eDES model consists of a gut and plasma compartments, in which the change in mass or concentration of glucose and insulin over time is described using coupled differential equation (full details provided in [9]). The reactions included in the model are controlled by rate parameters, which have been estimated and validated using OGTTs from multiple healthy populations [8].

In order to provide personalized simulation of glucose and insulin concentrations using the eDES model, we implemented the model selection approach that we have developed previously [9]. Briefly, the workflow reduces the number of model parameters to be estimated to provide an accurate and reliable description of individual postprandial glucose and insulin responses. To obtain the most sensitive, parsimonious and identifiable model parameter set, the following steps were undertaken. Firstly, a local parameter sensitivity analysis (LPSA) was performed to identify the most sensitive candidate parameters for estimation. Secondly, a set of all the possible combinations of 3 or more sensitive parameter candidates were generated. These were then fit on representative responses to oral glucose intake of individuals with different glucometabolic status (i.e. normal glucose metabolism (NGM), impaired fasting glucose (IFG), impaired glucose tolerant (IGT), both IFG and IGT (IFG&IGT), and T2DM); based on the American Diabetes Association (ADA) diagnosis criteria values [20], the most extreme responses of the Maastricht Study dataset, and on the largest and smallest response in the dataset by area under the glucose curve (Min, Max). The initial values for glucose and insulin in the eDES model were set to be equal to the t = 0 measurement of the response, and the set-point parameters $G_{b}^{pl}$ (basal plasma glucose) and $I_{b}^{pl}$ (basal plasma insulin) were also set to these initial values (t = 0). The candidate model with the lowest Akaike Information Criterion (AIC) score across the set of representative curves was selected as most parsimonious model, which was further evaluated for identifiability using Profile Likelihood Analysis (PLA) [21]. In this way, the model parameters to be estimated resulting from the model selection pertained to gut emptying (k1), insulin sensitivity (k5) or insulin secretion (k6 & k8) described in S1 Table. These results are in agreement with our previously reported findings in [9].

The parameter space of the Personalized eDES model is visualized by reducing the number of dimensions from the number of estimated parameters to two dimensions using principal component analysis (PCA). Prior to PCA, the parameter values were log-transformed and normalized to zero mean and unit standard deviation.

*Model fitting procedure.* The eDES model is fitted to time-series of glucose and insulin from the OGTT by providing as input to the model the corresponding t = 0 glucose and insulin measurements and then estimating the selected model parameters by minimizing the sum of squared residuals (SSR) between the model simulation and the measured time-series using a non-linear least squares solver (*lsqnonlin*). The output of the model is then the estimated glucose and insulin concentrations (as well as the estimated parameter values; not considered in this paper). The eDES model was implemented and analysed in MATLAB 2018b (The Mathworks, Inc., Natick, Massachusetts, United states).

#### Gradient boosting regression

As a data-driven model, we employed gradient boosting regression (GBR) models in order to allow non-linear relationships between the targets and the predictors [22, 23]. GBR works by combining the prediction of many different decision trees that were inferred sequentially by training the tree on the residual of the previous trees.

Relative feature importances are reported as the variance (MSE) reduction weighted by the proportion of samples reaching the node across all trees [24].

*Model fitting procedure.* A GBR model is fitted to each measurement of glucose and insulin concentration from the OGTT, independently. A wide range of phenotypic, demographic and lifestyle characteristics of the individuals (full details in supplementary material) were used as predictors in the gradient boosting regression models. The predictors were kept the same across all GBR models and no interactions between the predictors were considered. The objective function to be minimized in training was the mean squared error. The GBR models were trained in a nested cross-validation framework in order to provide an unbiased estimation of model performance as described by Cawley and Talbot [25]. Standardization of the numeric features was carried out within each fold to avoid introducing positive bias to the the estimates. Hyperparameter tuning was executed with the options described in S3 Table of the supplementary material. The performance estimates (see Performance estimates section below) were calculated in the outer 5-fold cross validation (CV), with the inner loop (also 5-fold CV) carrying out a limited hyperparameter search independently of the model training procedure. GBR models were implemented in python 3.5 using the XGBoost package [22].

#### Hybrid approach

In addition to applying the eDES and GBR models separately, we also employed a hybrid approach combing these two models. This hybrid model consists of sequentially joining the prediction of the eDES model and a GBR model. A schematic depicting the workflow and data usage of the hybrid approach is shown in S1 Fig.

*Model fitting procedure.* First, the eDES model is fitted to time-series of glucose and insulin from the OGTT. Then, the residuals in the eDES model predictions (i.e. difference between predicted and measured data-points) are calculated. Subsequently, GBR models are trained to predict the residuals from the phenotypic features independently per glucose and insulin measurement, thereby linking the two models together. Finally, the output of the hybrid approach is calculated by subtracting the GBR predicted residuals from the eDES model predictions. In this way, the GBR models are used to incorporate additional data (phenotypic features) to improve the eDES model estimated glucose-insulin trajectories.

#### Performance estimates

Model performances are evaluated by calculating the mean squared error of prediction (MSE) and the coefficient of determination (*R*^2^) for each model. The *R*^2^ and MSE in the model predictions are reported at each measurement time point of the OGTT and are compared for the different modeling scenarios.

#### Overview of modeling scenarios

A variety of modeling scenarios based on the eDES, GBR, and Hybrid approaches are evaluated and compared in terms of coefficient of determination (*R*^2^) and mean squared error of prediction (MSE). The modeling scenarios are selected in order to allow the comparison of mechanistic with data-driven as well as their sequential combinations. The final output in all scenarios are the predicted postprandial glucose and insulin concentrations of the OGTT. The modeling scenarios are detailed below.

- Reference eDES
- Reference GBR
- Hybrid I. (reference eDES + GBR)
- Personalized eDES model
- Hybrid II. (Personalized eDES + GBR)

In the Reference eDES scenario, the median glucose and insulin responses to the OGTT in the Maastricht Study are calculated by taking the median of the measurements per time-point of the OGTT across NGM individuals. Then, the eDES model is fitted on the median glucose and insulin responses. In contrast to the Reference eDES scenario, in the Personalized eDES scenario we fitted the eDES model on each individuals’ response. In this way, we can compare a population-level and personalized mechanistic models. The Reference GBR scenario consists of GBR models estimated for each time-point of the OGTT response to predict the corresponding glucose and insulin concentrations. In the Hybrid I scenario, a combined approach is used in which the residuals in the Reference eDES model predictions are used as targets for the GBR models, thereby sequentially joining the two approaches. For the Hybrid II scenario, the residuals in the Personalized eDES model predictions are used as targets for the GBR models.

## Results

A total of 2968 participants were included in the analysis after excluding individuals with missing measurements on 2 or more time points of the 7-point OGTT or missing baseline the (t = 0) or 2 the hours post-load (n = 483) measurement. Out of the 2968 included individuals 1436 (48%) were normoglycaemic, 906 (31%) demonstrated impaired glucose metabolism (IFG and/or IGT) and 625 (21%) had type 2 diabetes based on the OGTT. The characteristics of the study population are depicted in Table 1.

**Table 1:** Descriptive characteristics of the study population.

Characteristic	Total	NGM	IFG	IGT	IFG&IGT	T2DM
	n = 2968	n = 1436	n = 529	n = 134	n = 243	n = 625
Sex (%*male*)	51	39	65	37	55	68
Age (*years*)	59.8 (8.2)	57.6 (8.3)	59.7 (7.6)	60.9 (8.1)	63.3 (7.1)	63.1 (7.4)
BMI (*kg*/*m*^2^)	26.8 (4.3)	25.2 (3.5)	27.1 (3.9)	27.1 (4.1)	28.7 (4.5)	29.4 (4.7)
HbA1c (*mmol*/*mol*)	39.3 (6.9)	35.7 (3.7)	38.1 (4.1)	37.0 (4.0)	40.3 (4.7)	48.7 (7.0)
Matsuda index	4.0 (2.7)	5.2 (2.8)	3.4 (2.0)	3.4 (1.9)	2.5 (2.0)	2.3 (1.7)

NGM: normal glucose metabolism, IFG: impaired fasting glucose, IGT: impaired glucose tolerance, T2DM: type 2 diabetes mellitus

Matsuda index is calculated as $10000/{\left(G_{t0}*I_{t0}*\bar{G}*\bar{I}\right)}^{1/2}$, where *G*_*t*0_, $\bar{G}$ and *I*_*t*0_, $\bar{I}$ are the t = 0 and average glucose and insulin measurements of the 7-point OGTT in *mg*/*dl* and *μU*/*L*, respectively. Calculated when t = 0 and t = 120 measurements were available.

Values are means (standard deviations)

### Prediction task properties

**Fig 1:**
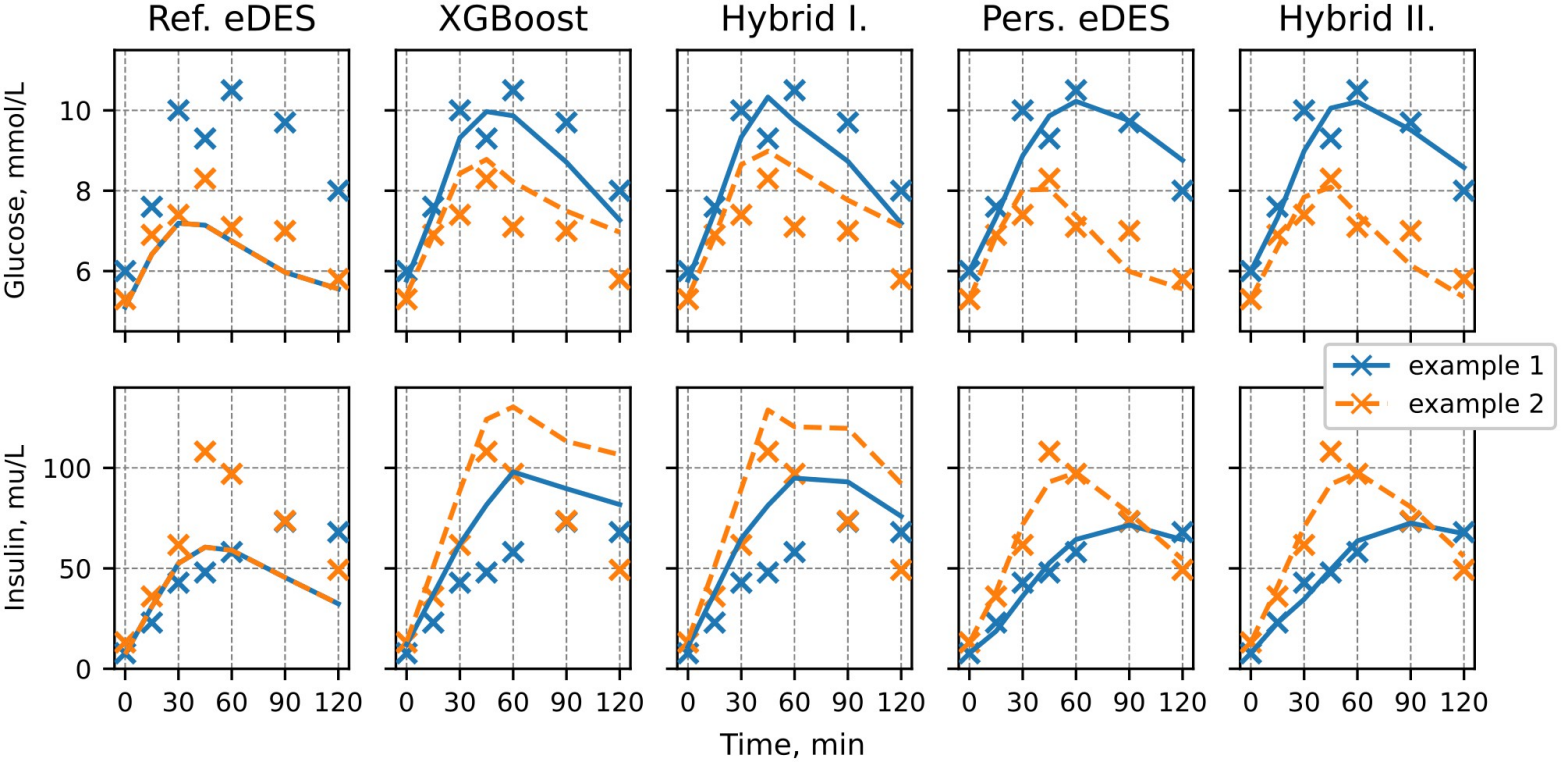
Examples of simulated OGTT responses of study participant using the modeling scenarios. Simulated glucose and insulin are depicted by blue and orange lines, respectively. The crosses denote the measured glucose and insulin concentrations.

To demonstrate the prediction task, an example of an individual’s OGTT response with corresponding simulation using the different modeling scenarios is provided in Fig 1.

The various modeling scenarios aimed to capture the measured data, denoted with black crosses. The discrepancy between the simulated glucose and insulin responses (in blue and orange, respectively) and the measured data-points, termed residuals, were summarized across individuals by calculating the error of prediction (MSE) and the explained variance (*R*^2^), and were used as comparison between the different modeling approaches in the following paragraph. As observed from this example, the Personalized eDES and Hybrid II approaches produced perfect predictions of the first (t = 0) glucose and insulin time-points. However, this resulted from the first measurement being supplied to the eDES model as initial values. Therefore, in these scenarios the performance measures (MSE, *R*^2^) were not reported.

### Modeling scenarios: Explained variance and error of prediction

**Fig 2:**
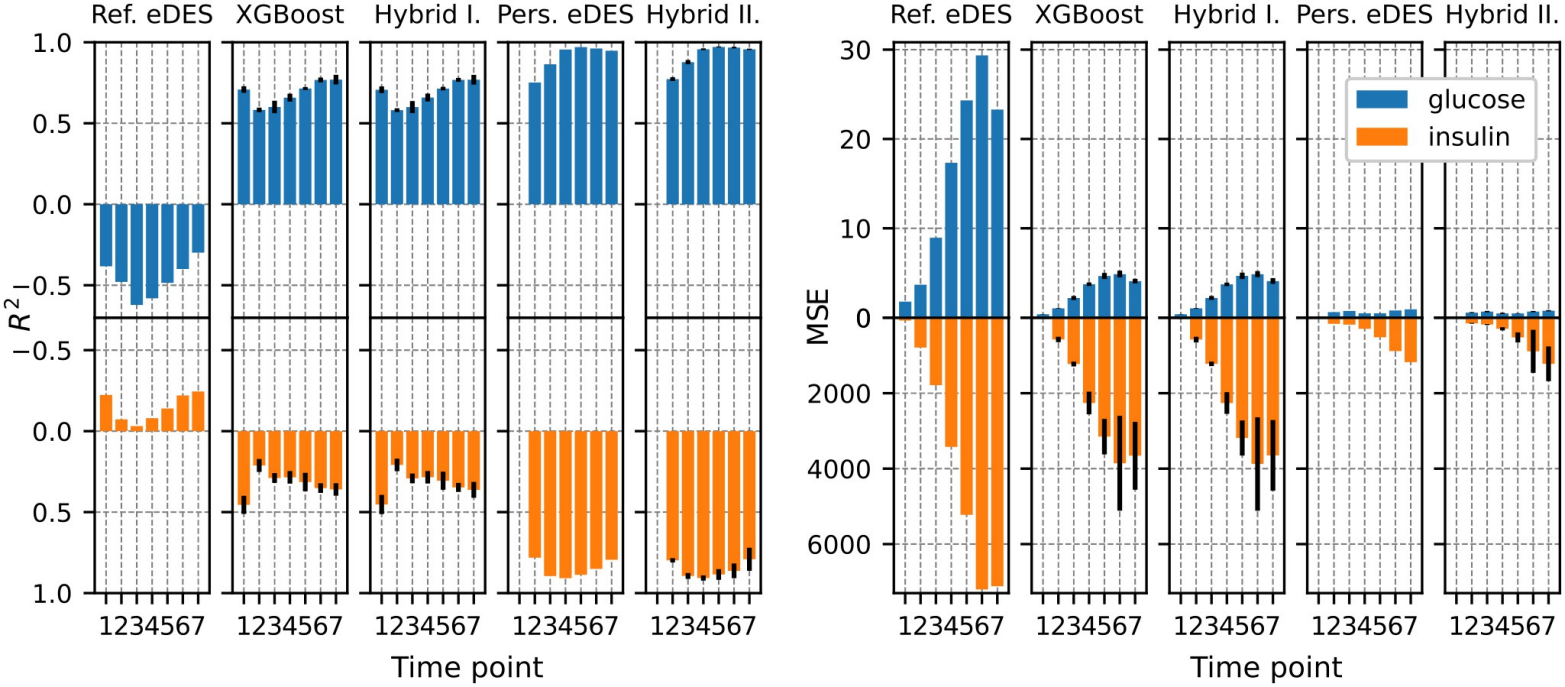
Explained variance (*R*^2^; panel A) and mean squared error (MSE; panel B) per glucose and insulin time-points following OGTT for the modeling scenarios.

An overall comparison of how well the different modeling scenarios (Reference eDES model, Reference GBR, Hybrid I, Personalized eDES model, and Hybrid II) perform in predicting the postprandial glucose and insulin levels is shown in Fig 2 and in S2 Fig and S2 Table. of the supplementary material. The *R*^2^ (Fig 2, panel A) and MSE (Fig 2, panel B) are provided for all the glucose and insulin time-points following the OGTT. The first glucose and insulin time-point for the Personalized eDES model and Hybrid II approach are not provided as these are used as initial values for glucose and insulin simulation via the eDES model.

For the Reference eDES scenario, the eDES model parameters k1, k5, k6, k8 were estimated on the NGM median OGTT response. The MSE ranged between 1.79 and 29.35 for glucose, 73.95 and 7205.55 for insulin, across all time-points (MSE and *R*^2^ values are provided in S2 Table). The MSE appeared to increase with respect to time, up until the 6th time-point. The *R*^2^ ranges between -0.62 and -0.30 for glucose, -0.03 and -0.25 for insulin, across all time-points. The negative explained variance indicates that the model is likely mis-specified for the majority of the OGTT responses. For the Reference GBR scenario, the GBR models were estimated for each time-point of the OGTT responses to predict the corresponding glucose and insulin concentrations using the features outlined in the methods section. The MSE ranges between 0.37 and 4.88 for glucose, 32.59 and 3857.80 for insulin, across all time-points. The MSE appears to increase with time, up until the 6th time-point. The *R*^2^ ranges between 0.58 and 0.77 for glucose, 0.21 and 0.46 for insulin, across all time-points. For the Hybrid I scenario, a hybrid approach was used in which the residuals in the individuals’ OGTT responses as predicted by the Reference eDES are used as targets for the GBR models. The MSE ranges between 0.38 and 4.87 for glucose, 32.73 and 3877.16 for insulin, across all time-points. The MSE appears to increase with time, up until the 6th time-points. The *R*^2^ ranges between 0.58 and 0.77 and 0.21 and 0.45 for glucose and insulin respectively, across all time-points. For the Personalized eDES model scenario, the eDES model is personalized through estimating eDES model parameters 1,5,6,8 for individual OGTT responses in the Maastricht Study. The MSE ranges between 0.49 and 0.77 for glucose, 161.03 and 1177.05 for insulin, across all time-points. The MSE appears to increase with time, up until the last (7th) time-point. The *R*^2^ ranges between 0.75 and 0.97 for glucose, 0.78 and 0.91 for insulin, across all time-points. For the Hybrid II scenario, the residuals in the individual OGTT responses obtained by the Personalized eDES models are used as targets for the GBR models. The MSE ranges between 0.47 and 0.77 for glucose, 148.30 and 1220.46 for insulin, across all time-points. The MSE appears to increase with time, up until the 7th time-points. The *R*^2^ ranges between 0.77 and 0.97 for glucose, 0.79 and 0.91 for insulin, across all time-points.

When comparing the different modeling scenarios, the Reference eDES model appeared to perform the worst across all glucose and insulin time-points, showing the largest MSE and the lowest *R*^2^. The data-driven XGBoost performed better than the Reference eDES model (81% and 42% decrease in MSE and 248% and 325% increase in *R*^2^ for glucose and insulin across all time-points). Combining the Reference eDES model with XGBoost (Hybrid I) resulted in an almost identical performance (81% and 42% decrease in MSE and 248% and 323% increase in *R*^2^ for glucose and insulin across all time-points). The Personalized eDES model performed much better than the Reference eDES model or the XGBoost model (96% and 85% decrease in MSE and 296% and 690% increase in *R*^2^ for glucose and insulin across all time-points when compared to the Reference eDES model). Combining the Personalized eDES model with XGBoost (Hybrid II) resulted in an almost identical performance (96% and 85% decrease in MSE and 298% and 693% increase *R*^2^ for glucose and insulin across all time-points).

### Modeling scenarios: Derivable information

**Fig 3:**
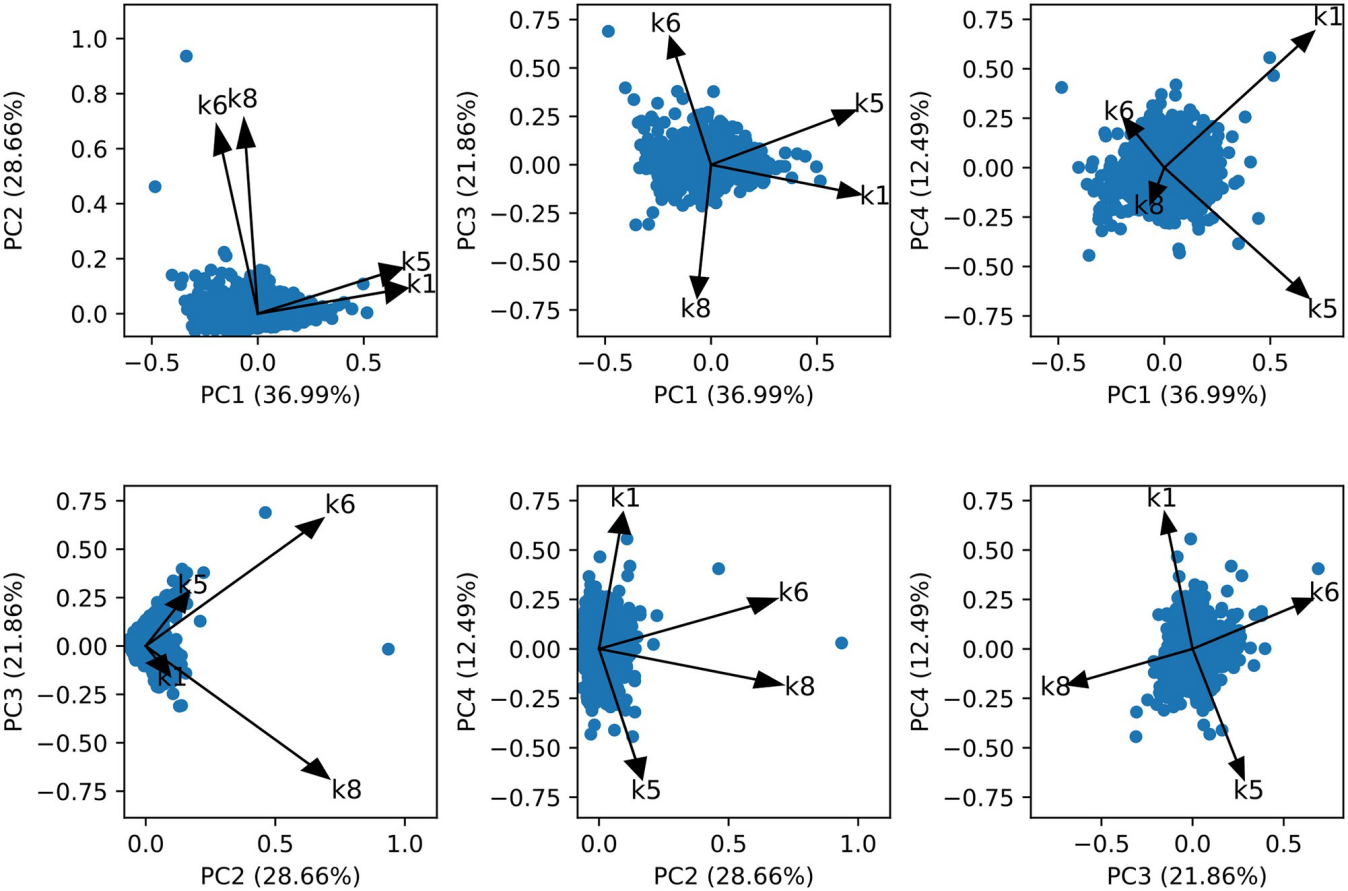
Individual parameter sets (n = 2968) in the parameter space of the Personalized eDES model. The parameter space spanning the direction of the estimated model parameters k1, k5, k6 and k8 is shown after dimensionality reduction via principal component analysis. Dots represent the estimated parameter sets of individuals from the Maastricht Study and arrows represent the loadings.

Both the mechanistic models and the data-driven models allow insight into factors underlying the OGTT responses. In the case of the mechanistic eDES model the tuned model parameters represent physiological properties such as gut emptying or insulin secretion. Whereas the data-driven XGBoost models are able to derive the features that were used to predict the responses. Since the Reference eDES model represents the population median response, the model parameters provide no distinction between individuals. The Personalized eDES models allow a comparison between individuals based on the estimated parameter values. In Fig 3, the individuals in the parameter space of the Personalized eDES models are shown after dimensionality reduction via PCA. The estimated parameter set of individuals can be used to quantify inter-individual variability. However, a detailed exploration of the eDES parameter space is outside of the scope of this work, as the aim here was to investigate novel features using an hybrid modeling approach. We previously examined these parameters in [9].

**Fig 4:**
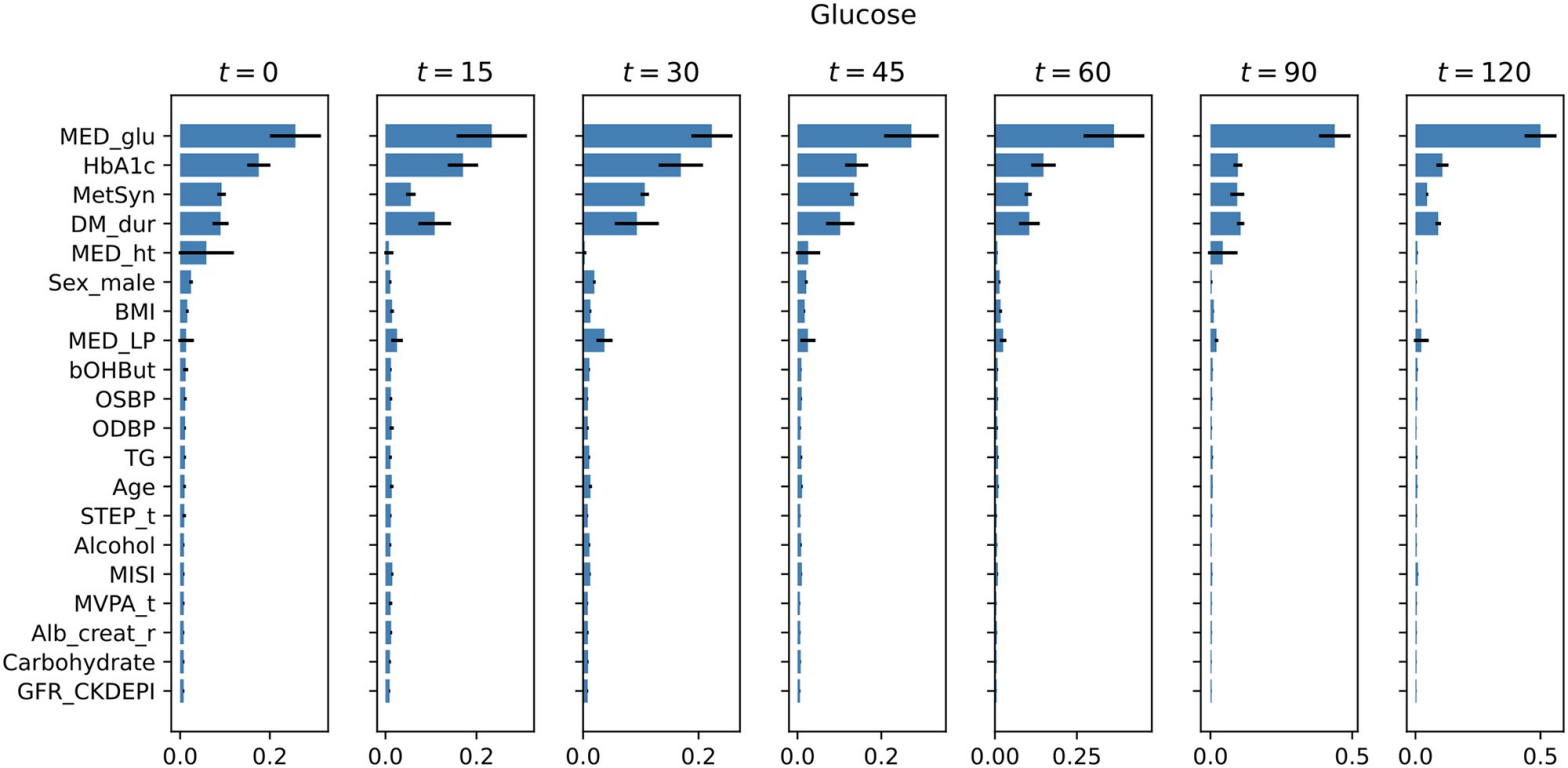
Relative feature importances in predicting the glucose concentration at the seven time points of the OGTT via the reference XGBoost models. The 20 most important features are in decreasing order by relative feature importance at t = 0. The relative feature importances (x-axes) are calculated as the variance (MSE) reduction weighted by the proportion of samples reaching the node across all trees. Error bars represent the standard deviation across cross-validation folds.

In the case of the XGBoost model, we can calculate the relative feature importances to derive the contribution of each feature to the model prediction. Fig 4 shows the relative feature importances of the 20 most important features (ordered by relative feature importance in the t = 0 model; the complete list is shown in S3 Fig) in the Reference XGBoost model predicting the postprandial glucose levels at all time-points. The relative feature importances per insulin time-point are shown in S4 Fig.

Across all time-points the most important features to the model predictions of glucose levels were whether the individual was taking glucose lowering medication (MED_glu), followed by HbA1c, presence of metabolic syndrome (MetSyn), and diabetes duration (DM_dur). In case of predicting the insulin levels, the most informative features were the presence of metabolic syndrome for all the time-points, BMI and the MISI score for time-points 2–6 and whether the individual was taking glucose lowering medication for time-points 2–4. Additional features appear to contribute to the model prediction in certain time-points, such as total cholesterol-to-HDL cholesterol ratio (Chol_r) at the 2nd time-point or the inflammation marker IL-8 at the 4th time-point.

The feature importances in the data-driven part of the Hybrid I scenario largely agree with those in case of the Reference XGBoost model. In the Hybrid II scenario, the Personalized eDES models showed good prediction performance, however the data-driven models had low predictive performance (S2 Table). Therefore, in contrast to the Hybrid I scenario, the interpretation of the feature importances in the Hybrid II scenario is hindered by a low explained variances observed in the data-driven models (S7 and S8 Figs).

## Discussion

In the present study, we investigated various modeling scenarios to predict glucose and insulin concentrations following an OGTT. We compared mechanistic modeling with data-driven models as well as the combination of the two approaches in order to evaluate predictive performance (measured as coefficient of determination, *R*^2^ and mean squared error of prediction, MSE) and to identify factors underlying inter-individual differences in the responses. In addition, we assessed whether the variance explained by a mechanistic model could be improved by explicitly accounting for additional factors such as body composition, lifestyle-related factors, and cardiometabolic health-related parameters, using a data-driven model. We showed that a mechanistic model, tuned on a large number of individual’s data from The Maastricht Study, was best suited to accurately capture individual OGTT responses, whereas, the combination with the data-driven model did not improve the prediction further.

The mechanistic glucose-insulin model used in this study employs time series data to estimate rate parameters related to glucose metabolism. The reference eDES model performed the worst of all the modeling scenarios for both *R*^2^ and MSE. In this approach, the estimated parameters represented a median OGTT response. Therefore, the large MSE and low *R*^2^ were not surprising, given the heterogeneity in individual OGTT responses. Nevertheless, the reference eDES model highlights the flaw in one-size fits all approaches, as well as providing us with a reference model that is not personalized. Using the individualization approach briefly described in the methods, we were able to accurately capture glucose and insulin concentrations using only 4 estimated model parameters. As expected, the model performance in terms of MSE and *R*^2^ was much better than the reference eDES model. In addition to being able to capture dynamic glucose and insulin responses, the mechanistic nature of this model provides quantitative information on the encoded processes linked to glucose metabolism. The individualization approach employed on the current data set resulted in the same sensitive, parsimonious, and identifiable parameter set as employed in our previous work, describing the rate constant of glucose appearance in the gut (k1), the rate constant of insulin-dependent glucose uptake (k5), the proportional rate constant of insulin secretion due to the difference in the actual plasma glucose level compared to baseline (k6), and the insulin secretion dependent on the rate of change in plasma glucose (k8). In part due to the eDES model structure, the estimated parameters are correlated to varying degree based on what processes they pertain to. For example, the association implied by the biplot of PC1 vs PC2 in Fig 3 is indicative of both k6 and k8 representing the rate of insulin secretion. A more detailed assessment of the eDES model structure is given in [9].

As opposed to mechanistic models of glucose homeostasis, data-driven models make no assumption about the underlying physiological properties of glucose homeostasis. Instead, the large amounts of heterogenous data are explored to find patterns associated with glucose and insulin levels in a hypothesis generating way. Glucose predictor models have been gaining ground in diabetes prevention and management with increasing success [15, 26]. Here, we trained gradient boosting regression models to predict glucose and insulin concentrations after an OGTT using the deep phenotyping features from the Maastricht Study. A nested cross-validation training scheme was used in order to provide unbiased performance estimates. The XGBoost models were able to explain a large part of the variance (S2 Table), however their predictive performance was considerably lower than the performance of the personalized eDES models. The explained variances for insulin concentrations were much lower, indicating that the features used in the models are more predictive of glucose, rather than insulin concentrations. In addition, the prediction error of the XGBoost model was much higher than the personalized eDES models. An advantage of the data-driven modeling used in this work is that a large number of features representing a wide range of characteristics can be used as predictors in the models, however in contrast to the mechanistic model, no time-dependency structures are taken into account. This independent time-point-wise modeling of the glucose and insulin concentrations disregard the correlation between glucose and insulin measurements at consecutive time-points of the OGTT in the same individual likely leading to less accurate predictions. Furthermore, as opposed to the eDES model, the XGBoost models did not account for the correlation between glucose and insulin measurements made in the same individual either. The feature importances of the XGBoost models indicate that the most predictive features of glucose concentrations (Fig 4) were well known measures of glucose homeostasis such as HbA1c or a prescription of diabetes medication leading to no novel insights. While the feature importances in the case of insulin predictions (S4 Fig) may show some interesting features to contribute to the predictions, however, the low coefficient of determination and high prediction error undermine their relevance.

In an attempt to evaluate whether the glucose and insulin predictions of the eDES model can be further improved by accounting for characteristics of individuals that are not explicitly modeled in the eDES model we combined the prediction of the eDES models with those of XGBoost models. The results of this proof-of-concept study showed that these hybrid models (models Hybrid I and Hybrid II) present little benefit in combining the two models indicated by the lack of improvement in either *R*^2^ or MSE. In the case of Hybrid I the data-driven part of the model is almost equivalent to the standalone XGBoost models (S2 Table, S5 and S6 Figs) implying there is no benefit to combining the predictions with the reference eDES model. While, in the case of Hybrid II the personalized eDES models performed very well and there seems to be no additional insight gained by adding the data-driven models to the prediction task (S2 Table, S7 and S8 Figs). Nevertheless, the performance of the hybrid models outlined in this paper should be considered in context of the limited scope of our paper. The lack of improvement in prediction performance may originate from the lack of time-dependency structure in the data-driven models, the choice of feature set but also from the features used in the data-driven model falling into the same causal pathway as the parameters of the eDES model. While the eDES model does not explicitly account for many of the features used in the data-driven modeling, it does account for high-level physiological processes that may encompass the information in those features, therefore leading to an issue of representations of the same causal pathways. For example, the estimated parameters of the eDES model in this work attribute the variance in the OGTT responses between individuals to differences in gut emptying (k1), insulin sensitivity (k5) or insulin secretion (k6 & k8). It is biologically plausible that the effect of certain features (e.g. physical activity) on the glucose and insulin predictions are already realized through one or a combination of parameter estimates of the eDES model (e.g. the value for k5; insulin sensitivity). Therefore, combining a mechanistic and a data-driven model in a sequential manner may not be appropriate, instead a parallel approach should be explored. In addition, the mechanistic model, that was employed in this study, was able to accurately describe the responses to standardized OGTTs. However, for complex meals containing varying amounts of macronutrients as well as meals in free-living conditions, the resulting glucose and insulin excursions may not be accurately captured. In such conditions, an approach combining a mechanistic model with a data-driven model may yield more informative results. A strength of this proof-of-concept study was the large and heterogeneous study population from The Maastricht Study. Individuals (n = 2968) with varying glycemic regulation including normoglycemia, prediabetes (impaired fasting glucose, impaired glucose tolerance or both conditions) as well as type 2 diabetes were present in the study population. Furthermore, the 7 time-point OGTT facilitates the observation of nuanced dynamics in the glucose and insulin profiles compared to the more prevalent 5 time-point test. In addition, the comprehensive profiling of the study participants included health behavior, cardiovascular health, musculoskeletal health, metabolic and demographic characteristics as well as body fat composition and biomarkers. The comprehensive phenotypic information of such a large number of individuals allowed the use of data-driven models to find patterns that may provide additional insight about the glucose regulation on top of the OGTT response.

## Conclusions

In the present study, we compared the predictive performance of mechanistic models with well-defined temporal dependencies (eDES), data-driven models with no temporal dependencies (XGBoost) and the sequential combination of the two (Hybrid I and Hybrid II). Our results suggest, that a 4 parameter model with appropriate temporal structure can vastly outperform a naive model built on a cross sectional phenotypic profile of an individual in predicting postprandial glucose and insulin concentrations. In addition, a sequential combination of a mechanistic and a data-driven approach may not be suitable when studying the underlying factors of inter-individual variance. Nevertheless, we show that the eDES model is especially convenient when temporal dynamics in the glucose and insulin responses are to be quantified. Furthermore, the findings presented in this work corroborate our previous results indicating that that the personalized eDES models are suitable to capture nuanced dynamics in the responses.

## Supporting information

S1 Fig Schematic of the hybrid approach. The eDES model is fitted to the glucose and insulin time series of the OGTT response. Then, the residuals in the predictions (i.e. difference between predicted and measured data-points) are calculated. Subsequently, an XGBoost model is trained per glucose and insulin time-point to predict the residuals of the eDES models by incorporating the phenotypic features. The output of the hybrid approach is generated by subtracting the XGBoost predicted residuals from the eDES model predictions.
(TIFF)

S2 Fig Measured versus predicted glucose and insulin concentrations at the time points (columns) of the OGTT in the case of the modelling scenarios (rows). (TIFF)

S3 Fig Relative feature importances in the Ref. GBR scenario predicting the postprandial glucose levels. Feature importances are in decreasing order by relative feature importance at t = 0. The relative feature importances (x-axes) are calculated as the variance (MSE) reduction weighted by the proportion of samples reaching the node across all trees. Error bars represent the standard deviation across CV folds.
(TIFF)

S4 Fig Relative feature importances in the Ref. GBR scenario predicting the postprandial insulin levels. Feature importances are in decreasing order by relative feature importance at t = 0. The relative feature importances (x-axes) are calculated as the variance (MSE) reduction weighted by the proportion of samples reaching the node across all trees. Error bars represent the standard deviation across CV folds.
(TIFF)

S5 Fig Relative feature importances in the GBR models of the Hybrid I scenario predicting the postprandial glucose levels. Feature importances are in decreasing order by relative feature importance at t = 0. The relative feature importances (x-axes) are calculated as the variance (MSE) reduction weighted by the proportion of samples reaching the node across all trees. Error bars represent the standard deviation across CV folds.
(TIFF)

S6 Fig Relative feature importances in the GBR models of the Hybrid I scenario predicting the postprandial insulin levels. Feature importances are in decreasing order by relative feature importance at t = 0. The relative feature importances (x-axes) are calculated as the variance (MSE) reduction weighted by the proportion of samples reaching the node across all trees. Error bars represent the standard deviation across CV folds.
(TIFF)

S7 Fig Relative feature importances in the GBR models of the Hybrid II scenario predicting the postprandial glucose levels. Feature importances are in decreasing order by relative feature importance at t = 0. The relative feature importances (x-axes) are calculated as the variance (MSE) reduction weighted by the proportion of samples reaching the node across all trees. Error bars represent the standard deviation across CV folds.
(TIFF)

S8 Fig Relative feature importances in the GBR models of the Hybrid II scenario predicting the postprandial insulin levels. Feature importances are in decreasing order by relative feature importance at t = 0. The relative feature importances (x-axes) are calculated as the variance (MSE) reduction weighted by the proportion of samples reaching the node across all trees. Error bars represent the standard deviation across CV folds.
(TIFF)

S1 Appendix List of phenotypic variables. (PDF)

S1 Table Explanation of estimated eDES model parameters. (PDF)

S2 Table R^2^ and MSE of prediction per time point of glucose and insulin in the OGTT in the modelling scenarios. (PDF)

S3 Table Hyperparameter search settings of the XGBoost models. (PDF)
