# Supplementary figures and images for: Quantifying postprandial glucose responses using a hybrid modeling approach: Combining mechanistic and data-driven models in The Maastricht Study
Source: PLoS One. 2023 Jul 27;18(7):e0285820. doi: 10.1371/journal.pone.0285820 (PMC10374070; doi:10.1371/journal.pone.0285820)

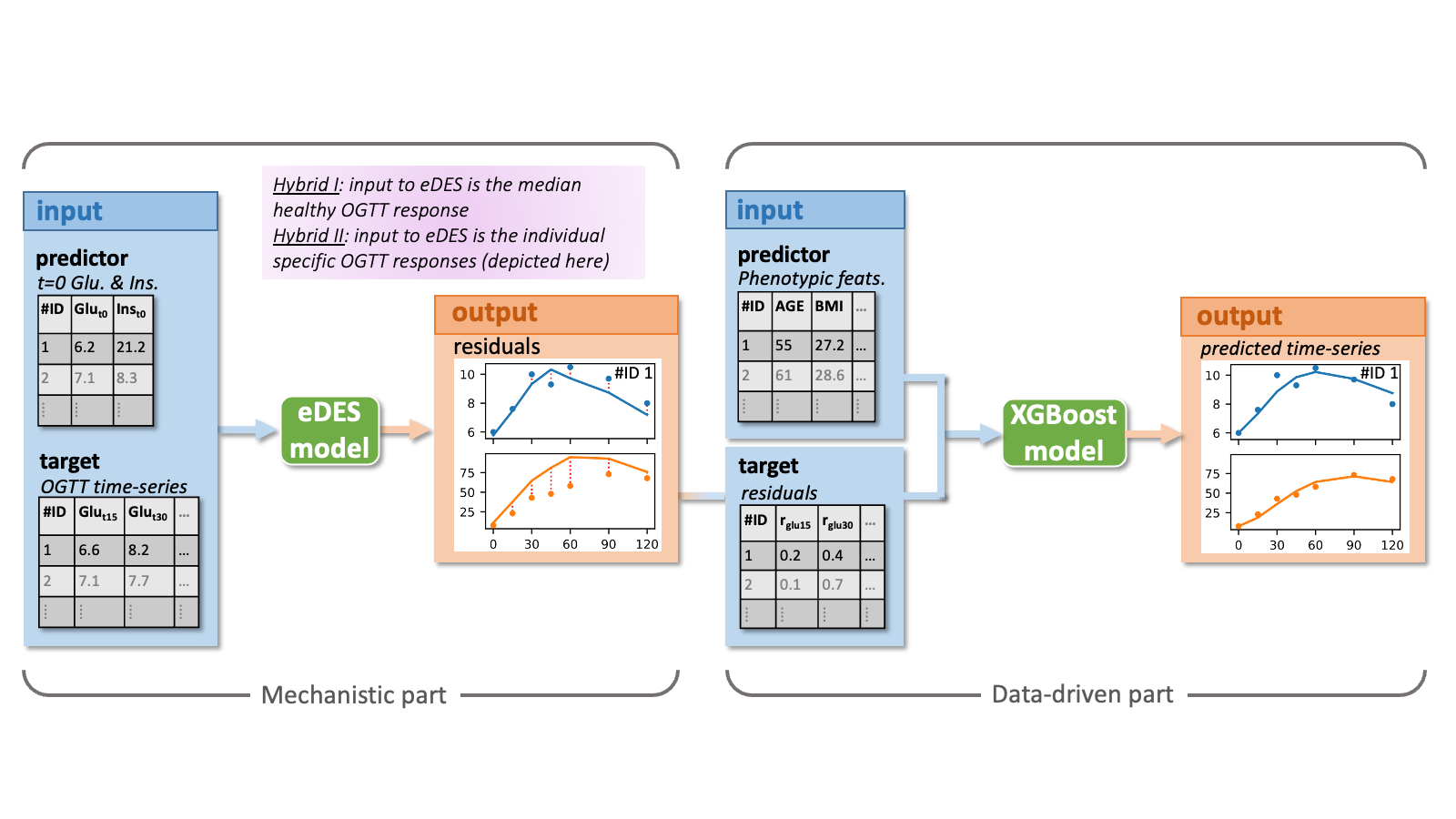

Supplement: S1 Fig — The eDES model is fitted to the glucose and insulin time series of the OGTT response. Then, the residuals in the predictions (i.e. difference between predicted and measured data-points) are calculated. Subsequently, an XGBoost model is trained per glucose and insulin time-point to predict the residuals of the eDES models by incorporating the phenotypic features. The output of the hybrid approach is generated by subtracting the XGBoost predicted residuals from the eDES model predictions. (TIFF) [file pone.0285820.s001.tiff]

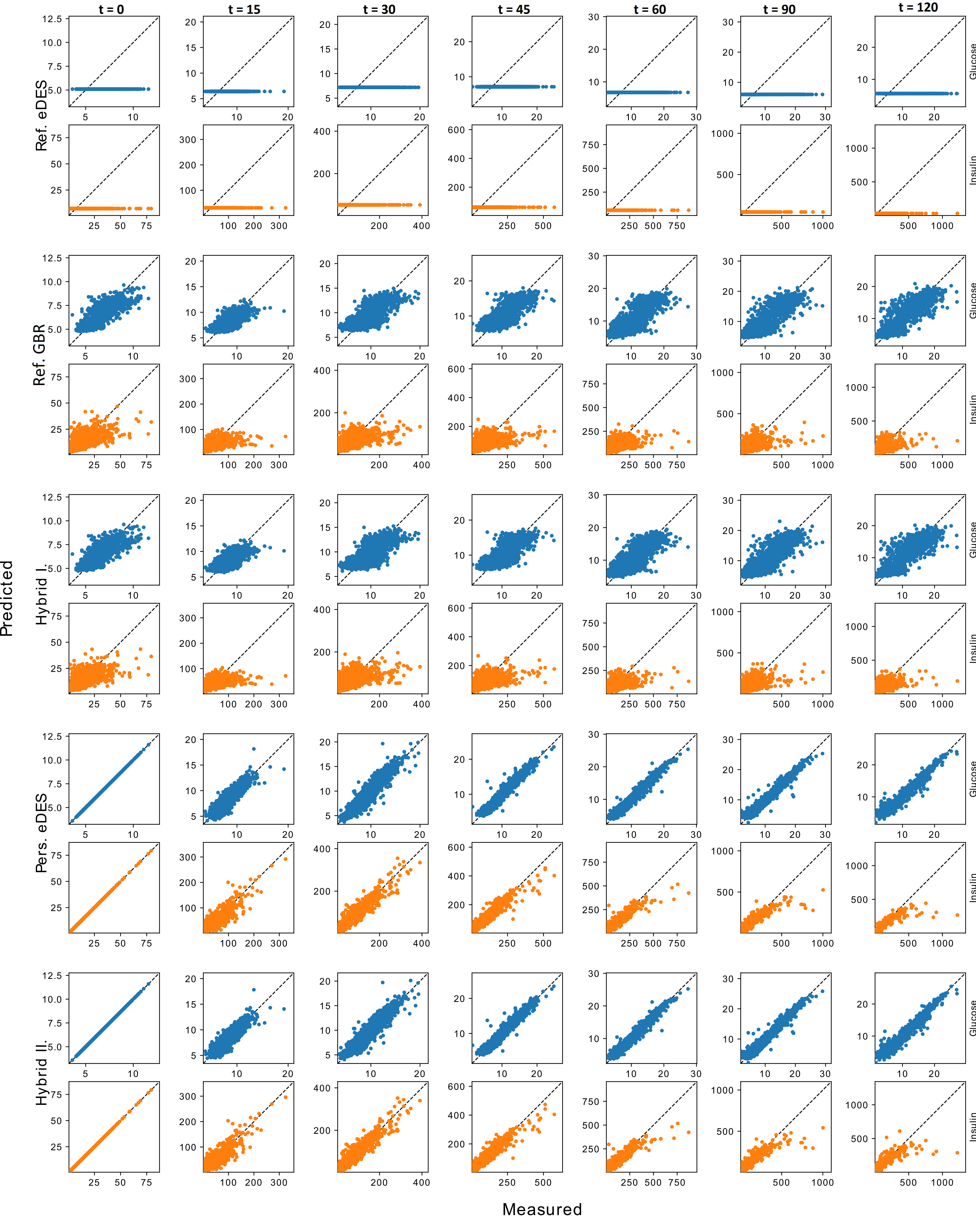

Supplement: S2 Fig — (TIFF) [file pone.0285820.s002.tiff]

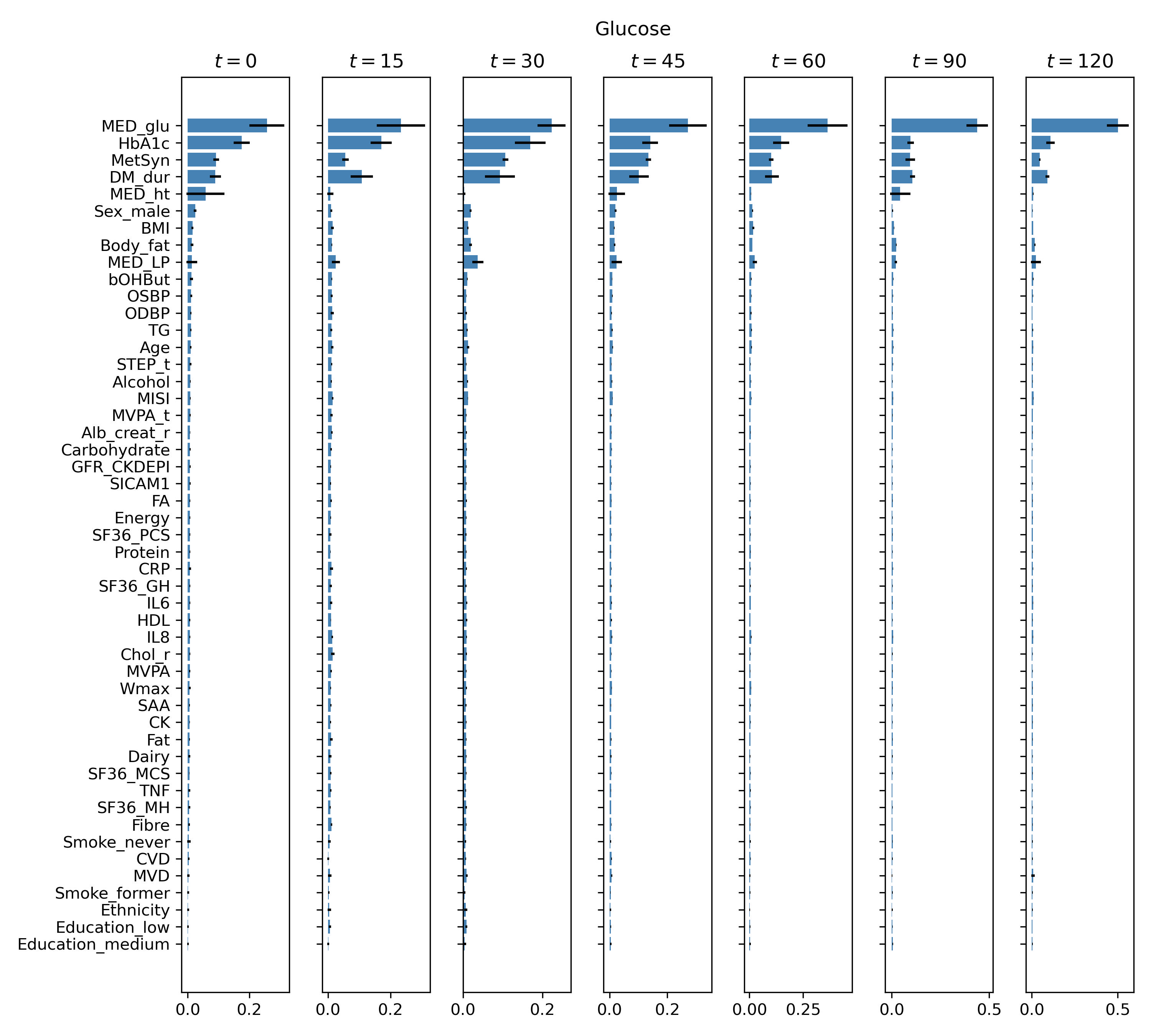

Supplement: S3 Fig — Feature importances are in decreasing order by relative feature importance at t = 0. The relative feature importances (x-axes) are calculated as the variance (MSE) reduction weighted by the proportion of samples reaching the node across all trees. Error bars represent the standard deviation across CV folds. (TIFF) [file pone.0285820.s003.tiff]

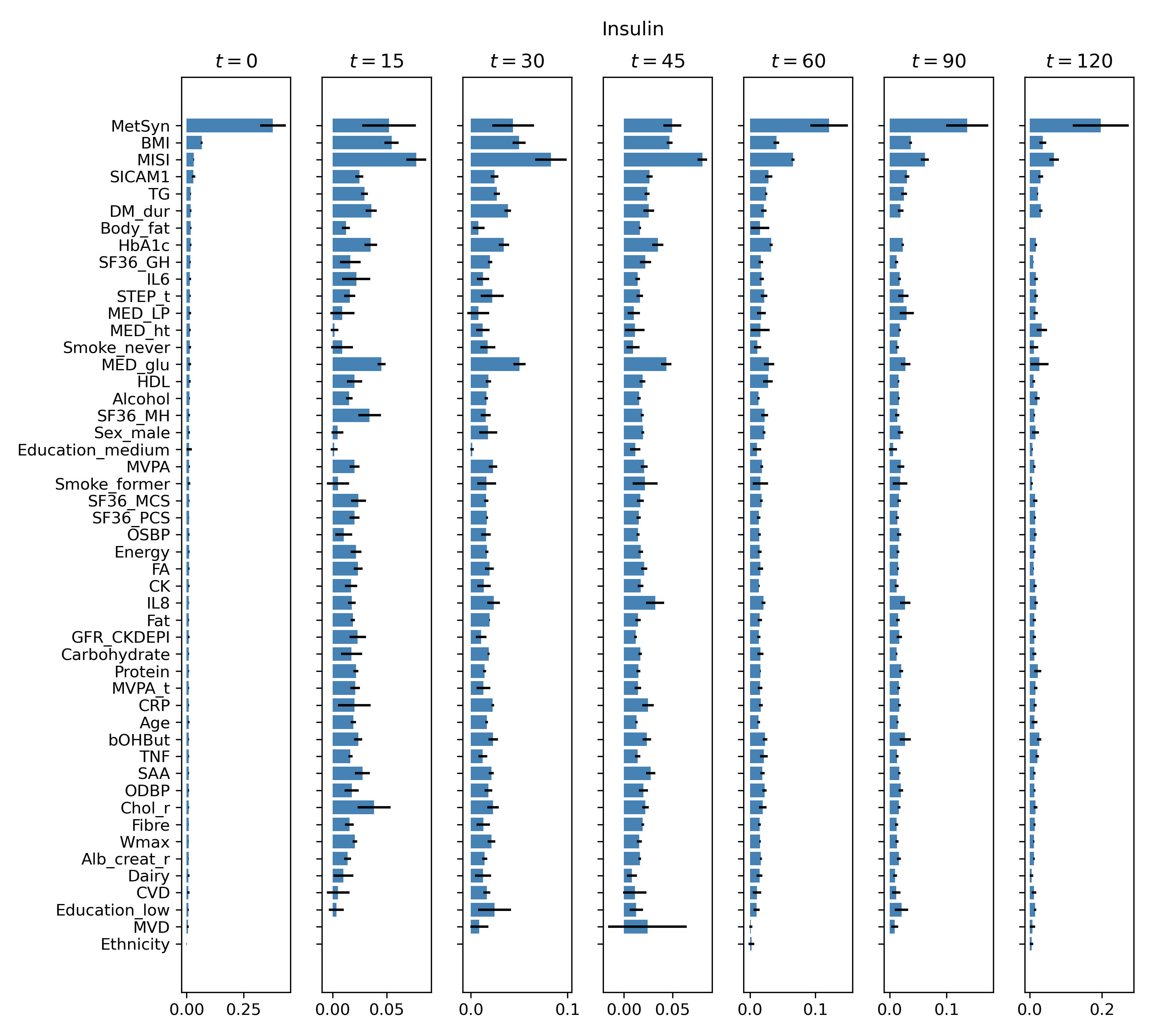

Supplement: S4 Fig — Feature importances are in decreasing order by relative feature importance at t = 0. The relative feature importances (x-axes) are calculated as the variance (MSE) reduction weighted by the proportion of samples reaching the node across all trees. Error bars represent the standard deviation across CV folds. (TIFF) [file pone.0285820.s004.tiff]

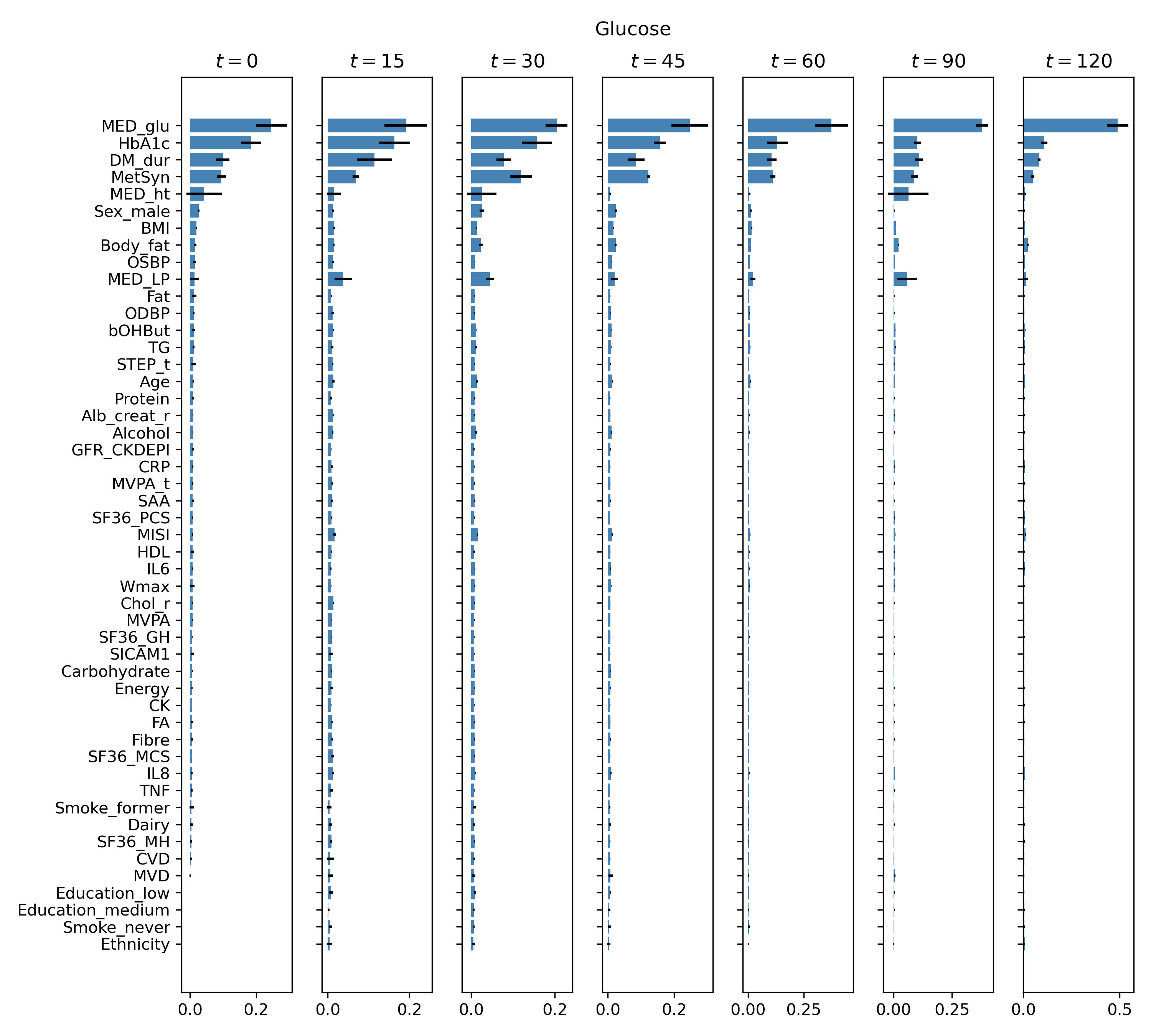

Supplement: S5 Fig — Feature importances are in decreasing order by relative feature importance at t = 0. The relative feature importances (x-axes) are calculated as the variance (MSE) reduction weighted by the proportion of samples reaching the node across all trees. Error bars represent the standard deviation across CV folds. (TIFF) [file pone.0285820.s005.tiff]

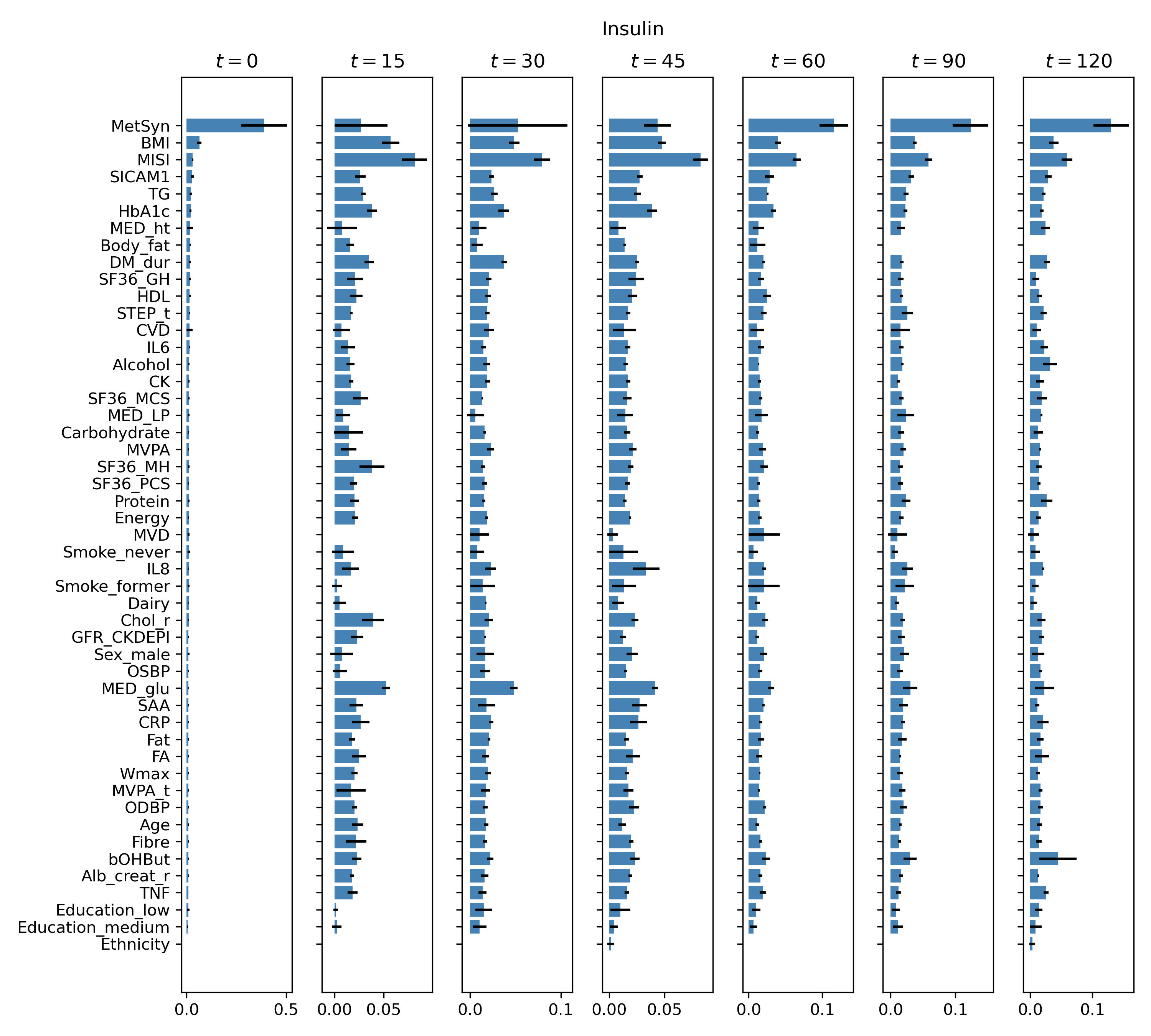

Supplement: S6 Fig — Feature importances are in decreasing order by relative feature importance at t = 0. The relative feature importances (x-axes) are calculated as the variance (MSE) reduction weighted by the proportion of samples reaching the node across all trees. Error bars represent the standard deviation across CV folds. (TIFF) [file pone.0285820.s006.tiff]

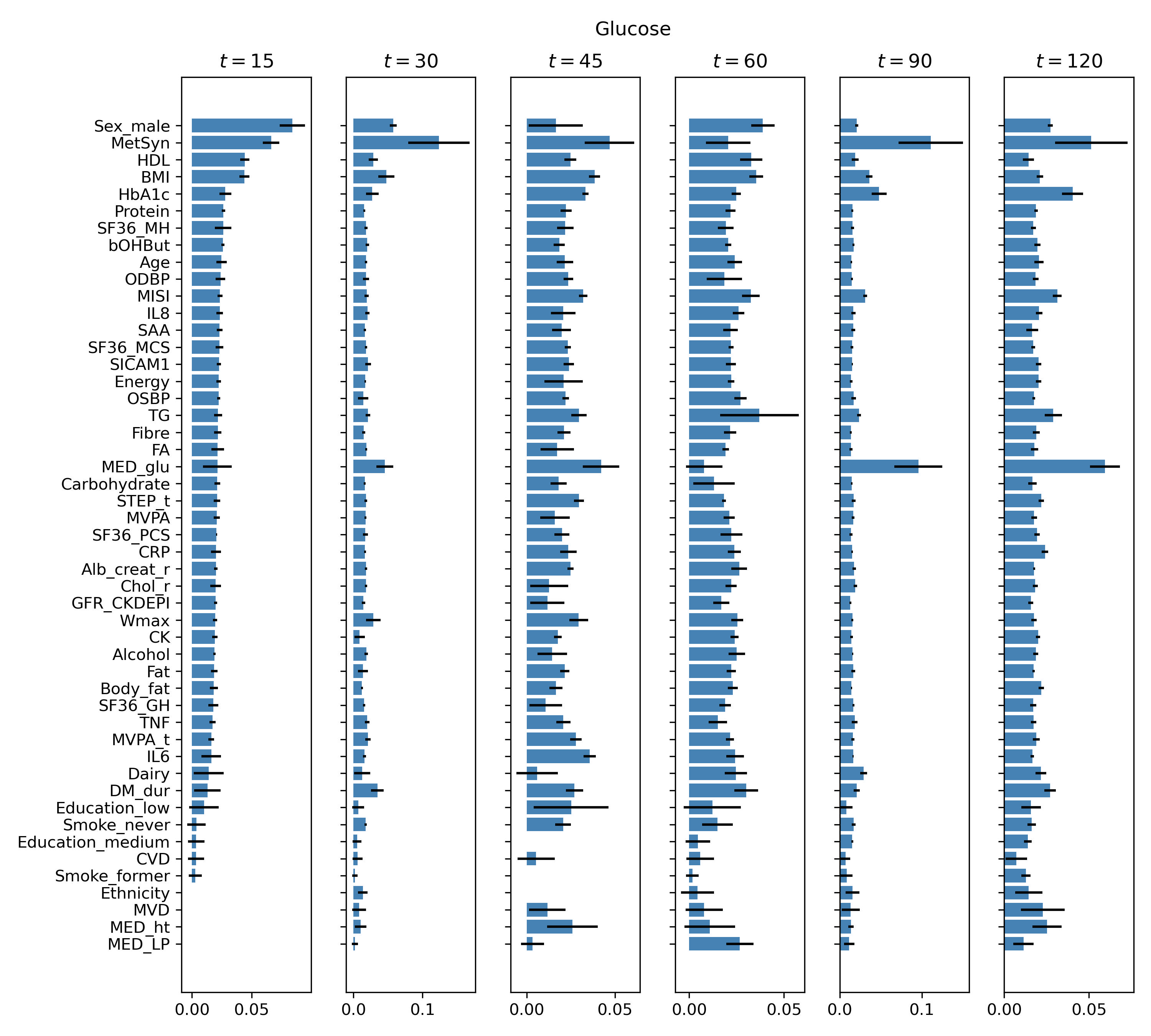

Supplement: S7 Fig — Feature importances are in decreasing order by relative feature importance at t = 0. The relative feature importances (x-axes) are calculated as the variance (MSE) reduction weighted by the proportion of samples reaching the node across all trees. Error bars represent the standard deviation across CV folds. (TIFF) [file pone.0285820.s007.tiff]

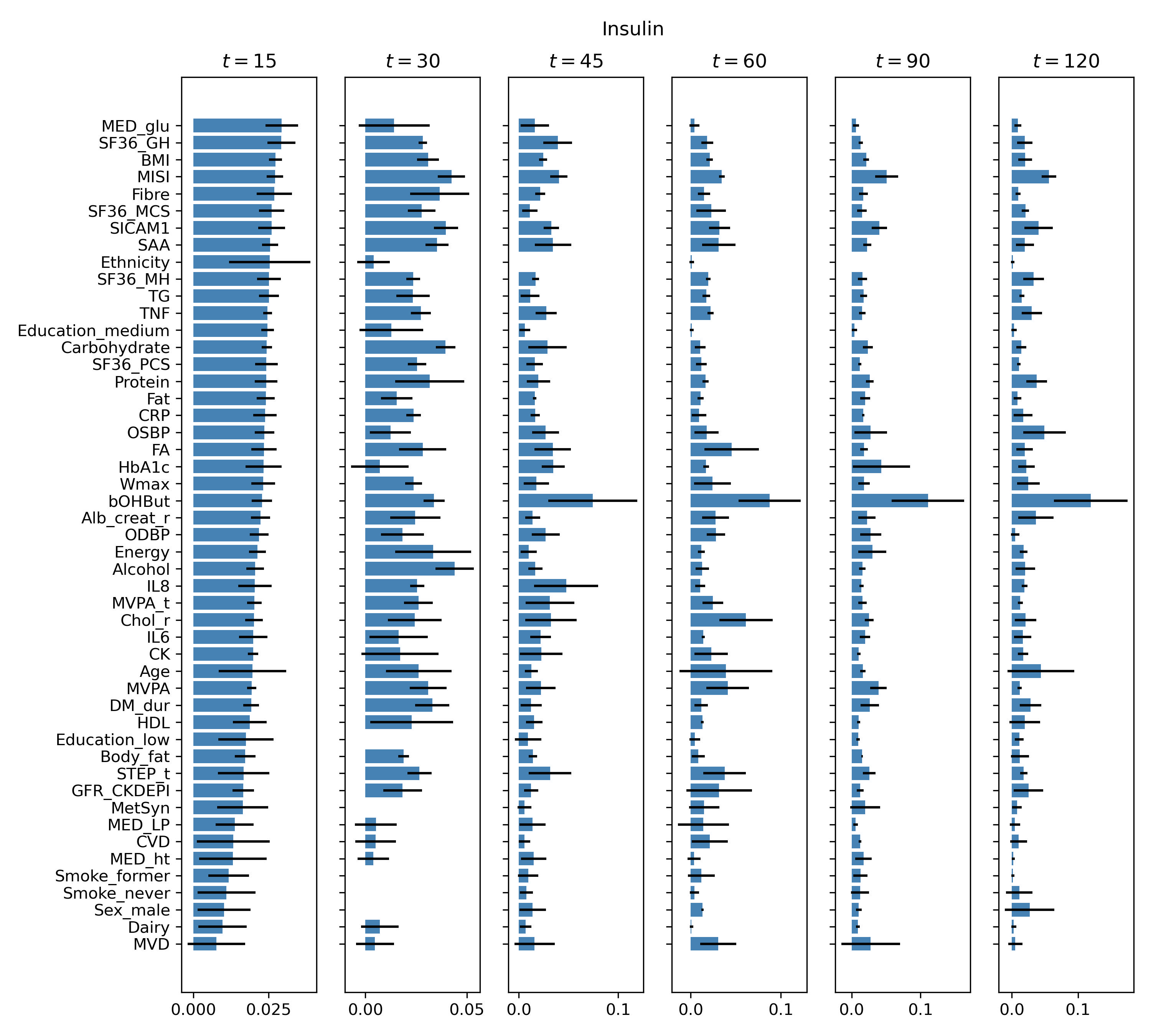

Supplement: S8 Fig — Feature importances are in decreasing order by relative feature importance at t = 0. The relative feature importances (x-axes) are calculated as the variance (MSE) reduction weighted by the proportion of samples reaching the node across all trees. Error bars represent the standard deviation across CV folds. (TIFF) [file pone.0285820.s008.tiff]
