# Supplementary material for: Quantifying postprandial glucose responses using a hybrid modeling approach: Combining mechanistic and data-driven models in The Maastricht Study
Source: PLoS One. 2023 Jul 27;18(7):e0285820. doi: 10.1371/journal.pone.0285820 (PMC10374070; doi:10.1371/journal.pone.0285820)
Supplement: S1 Appendix — (PDF) [file pone.0285820.s009.pdf]

S1 Appendix: List of phenotypic variables

Anthropometrics

Age: Age at visit (years), Sex_male: Sex, BMI: Body Mass Index, Body_fat: Total body fat percentage (%), Smoke_never/former: Smoking status (3 categories)

Demographics

Ethnicity: Ethnicity, Education_low/medium: Educational level categories

Health behaviour

MVPA_t: mean MVPA wake minutes per day total, STEP_t: mean step wake minutes per day total, Wmax: Estimated maximal power output adjusted for body weight (Wkg^{-1}), MVPA: moderate to vigorous physical activity per week ($hours/week$), Alcohol: Alcohol total (g/day), Protein: Protein total (g/day), Energy: Energy (KJ), Carbohydrate: Carbohydrates total (g/day), Fat: Fat total (g/day), Fibre: Dietary fibre total (g/day), Dairy: dairy products with probiotics (g)

Medication

MED_glu: Glucose-lowering medication (oral only), Glucose-lowering medication or insulin, MED_HT: Blood pressure lowering medication (all types),MED_LP: Lipid-modifying medication

Cardiovascular health

CVD: history of cardiovascular disease, ODBP: Diastolic Blood Pressure, OSBP: Systolic Blood Pressure

Metabolic health

Matsuda index, HIRI index ($(mmol/L)(pmol/L)(hour)^2$), c-peptidogenic index t30, Fasting plasma Glucose ($mmol/L$), glucose tolerance status (WHO), HOMA2, MetSyn: metabolic syndrome, MVD: Microvacular disease, DM_dur: Duration of type 2 diabetes in years, MISI: MISI index ($umol/L/min/pmol/L$),

Biomarkers

HDL: Serum HDL cholesterol ($mmol/l$), Chol_r: Total cholesterol-to-HDL cholesterol ratio, HbA1c: HbA1c ($mmol/mol$), Alb_creat_r: Albumin-creatinine ratio ($g/molcreatinine$), bOHBut: 3-hydroxybutyrate ($mmol/l$), CK: Creatine kinase in serum (U/L), GFR_CKDEPI: Glomerular filtration rate ($ml/min/1.73m^2$), CKDEPI using serum creatinine, FA: Total fatty acids ($mmol/l$), TG: Serum triglycerides ($mmol/l$)

Inflammation markers

CRP: C-reactive protein (g/ml), IL6: Human interleukin-6 (pg/ml), IL8: Human interleukin-8 (pg/ml), SICAM1: Soluble intercellular adhesion molecule-1 (ng/ml), TNF: Human tumor necrosis factor alpha (pg/ml), SAA: Serum amyloid A (g/ml)

Self reported quality of life

SF36_GH: SF36 general health, SF36_MCS: SF36 Mental component summary score, SF36_MH: SF36 mental health,SF36_PCS: SF36 Physical component summary score
