# Supplementary material for: Quantifying postprandial glucose responses using a hybrid modeling approach: Combining mechanistic and data-driven models in The Maastricht Study
Source: PLoS One. 2023 Jul 27;18(7):e0285820. doi: 10.1371/journal.pone.0285820 (PMC10374070; doi:10.1371/journal.pone.0285820)
Supplement: S1 Table — (PDF) [file pone.0285820.s010.pdf]

Explanation of estimated eDES model parameters

Table S1: *

Name	Description	Units
$k1$	Rate constant of glucose appearance in the gut	1/min
$k5$	Rate constant of insulin-dependent glucose uptake	1/min
$k6$	Rate constant of ΔG dependant insulin production	1/min
$k8$	Rate constant of $\frac{dG}{dt}$ dependant insulin production	1/min

where G is plasma glucose concentration. For details see [1].

References

[1] Balázs Erdős et al. "Personalized computational model quantifies heterogeneity in postprandial responses to oral glucose challenge". In: *PLoS computational biology* 17.3 (2021), e1008852.
