# Supplementary material for: Quantifying postprandial glucose responses using a hybrid modeling approach: Combining mechanistic and data-driven models in The Maastricht Study
Source: PLoS One. 2023 Jul 27;18(7):e0285820. doi: 10.1371/journal.pone.0285820 (PMC10374070; doi:10.1371/journal.pone.0285820)
Supplement: S2 Table — (PDF) [file pone.0285820.s011.pdf]

R^2 and MSE of prediction per time point of glucose and insulin in the OGTT in the modelling scenarios

		Ref. eDES		Pers. eDES		Ref. GBR		Hybrid I.		Hybrid II.	
M	TP	R^2	MSE	R^2	MSE	R^2	MSE	R^2	MSE	R^2	MSE
Glucose	1	-0.38	1.79	-	-	0.71 (0.02)	0.37 (0.02)	0.71 (0.02)	0.38 (0.02)	-	-
	2	-0.48	3.69	0.75	0.62	0.58 (0.01)	1.04 (0.03)	0.58 (0.01)	1.04 (0.04)	0.77 (0.01)	0.56 (0.05)
	3	-0.62	8.95	0.86	0.75	0.60 (0.04)	2.20 (0.23)	0.60 (0.04)	2.20 (0.22)	0.88 (0.01)	0.68 (0.08)
	4	-0.58	17.33	0.96	0.49	0.66 (0.02)	3.74 (0.20)	0.66 (0.02)	3.73 (0.20)	0.96 (0.01)	0.47 (0.08)
	5	-0.49	24.32	0.97	0.49	0.71 (0.01)	4.66 (0.34)	0.71 (0.01)	4.68 (0.36)	0.97 (0.00)	0.47 (0.03)
	6	-0.40	29.35	0.96	0.80	0.77 (0.01)	4.88 (0.38)	0.77 (0.01)	4.87 (0.34)	0.97 (0.00)	0.68 (0.07)
	7	-0.30	23.29	0.95	0.94	0.77 (0.03)	4.09 (0.26)	0.77 (0.03)	4.10 (0.32)	0.96 (0.00)	0.77 (0.07)
Insulin	1	-0.22	73.95	-	-	0.46 (0.06)	32.59 (2.50)	0.45 (0.06)	32.73 (2.78)	-	-
	2	-0.07	789.97	0.78	161.03	0.21 (0.04)	585.75 (72.72)	0.21 (0.04)	578.41 (75.55)	0.80 (0.01)	148.30 (10.93)
	3	-0.03	1788.02	0.89	182.72	0.29 (0.03)	1227.06 (63.90)	0.29 (0.03)	1222.82 (59.75)	0.89 (0.02)	179.72 (13.84)
	4	-0.08	3422.29	0.91	292.46	0.29 (0.04)	2259.30 (299.14)	0.28 (0.04)	2260.18 (287.06)	0.91 (0.02)	292.76 (41.76)
	5	-0.14	5224.25	0.89	517.77	0.31 (0.06)	3152.21 (470.09)	0.31 (0.06)	3187.93 (464.78)	0.88 (0.03)	521.00 (133.79)
	6	-0.22	7205.55	0.85	882.94	0.35 (0.03)	3857.80 (1256.49)	0.35 (0.03)	3877.16 (1238.39)	0.86 (0.05)	890.21 (570.07)
	7	-0.25	7120.25	0.79	1177.05	0.36 (0.04)	3659.74 (899.36)	0.36 (0.05)	3647.84 (936.59)	0.79 (0.07)	1220.46 (460.50)
