# Supplementary material for: Quantifying postprandial glucose responses using a hybrid modeling approach: Combining mechanistic and data-driven models in The Maastricht Study
Source: PLoS One. 2023 Jul 27;18(7):e0285820. doi: 10.1371/journal.pone.0285820 (PMC10374070; doi:10.1371/journal.pone.0285820)
Supplement: S3 Table — (PDF) [file pone.0285820.s012.pdf]

Hyperparameter search settings of the XGBoost models

parameter	range	number of equidistant samples
n_estimators	150-200	10
max_depth	2-15	7
learning_rate	0.01-0.05	11
subsample	0.7-0.9	21
colsample_bytree	0.48-0.98	11
min_child_weight	1-9	8
